# Totally tubeless percutaneous nephrolithotomy: Is it safe?

**Published:** 2009

**Authors:** T. J. Nirmal, Adhikary Samiran

**Affiliations:** Department of Urology, Christian Medical College, Vellore, India E-mail: nirmaltj@cmcvellore.ac.in

## SUMMARY

This was a randomized, controlled trial comparing the outcomes of percutaneous nephrolithotomy (PCNL) with and without a nephrostomy drain.[1] A sample size of 25 patients in each group was calculated based on data from a retrospective audit. The mean stone size was 21.6 vs. 17.5 mm. Patients underwent PCNL by a standard technique and were randomized if there was no significant bleeding, residual stone load, and if the pelvicalyceal system remained intact at the end of the procedure. In Group 1, a 26Fr nephrostomy tube was placed; for patients in Group 2 the puncture wound was sutured primarily. Analysis of the results showed no significant difference between the two groups with respect to analgesic requirement, drop in hematocrit, or infection rates. However, the mean length of hospital stay was significantly shorter in the tubeless group. The authors conclude that totally tubeless PCNL is safe in selected patients resulting in a significant decrease in hospital stay.

## COMMENTS

PCNL is accepted as the procedure of choice for the treatment of large or complex renal calculi.[2] Since its introduction in 1976,[3] the PCNL technique has been steadily refined and improved. Following the procedure, percutaneous drainage of the kidney is usually advocated. The nephrostomy tube is used for several purposes such as tamponade of bleeding from the track, proper drainage of urine, and access to the collecting system if a second look PCNL is required.[4]

Several recent studies have explored other alternative drainages systems like internal ureteral stent, small bore nephrostomy, or externalized ureteral stent in select patients with favorable outcomes.[5,6] However, reports on the totally tubeless approach have been sparse.

The major drawback of this trial has been the bias in patient selection, which was influenced by the surgeon's perception of the need for a nephrostomy tube. Cases with a small stone size and an uneventful procedure were selected for randomization. A stone of 10–20 mm is amenable to treatment with shockwave lithotripsy unless factors of stone composition, location, or renal anatomy suggest a more optimal outcome with an invasive modality like PCNL. In this trial, the tubeless group recorded a mean stone size of 17.5 mm only and the indication for PCNL is unclear. Hence, it is difficult to extrapolate the results of this trial for larger, more complex stones. Moreover, once the nephrostomy is placed, the caregiver is inevitably unblinded. This may lead to bias while determining parameters such as requirement of analgesia, incidence of infection, duration of hospital stay, etc., that favor the tubeless group. Other factors contributing to patient morbidity such as the number of punctures, choice of calyx, size of the Amplatz sheath, etc., have not been discussed. With the advent of tubeless PCNL, various substances have been tried to seal the nephrostomy tract in order to control tract bleeding and urinary extravasation, e.g., Fibrin glue, surgicel, gelatin matrix, etc. However, one positive outcome of this study was that primary suturing of the nephrostomy tract without use of any sealants seemed adequate.

This study manages to highlight the lack of standardization with regard to kidney drainage and the precise indication for tubeless PCNL. The totally tubeless procedure appears to be safe only in a highly select group of patients and results in a significant decrease in hospital stay.[1,5] However, this study fails to solve the dilemma concerning its real indication and benefit.
